# Sleep/wake regularity influences how stress shapes executive function

**DOI:** 10.3389/frsle.2024.1359723

**Published:** 2024-04-03

**Authors:** Gabriel R. Gilmore, Anna L. Smith, Fallon B. Dickinson, Alexandra D. Crosswell, Wendy Berry Mendes, Lauren N. Whitehurst

**Affiliations:** ^1^Department of Psychology, University of Kentucky, Lexington, KY, United States; ^2^Department of Statistics, University of Kentucky, Lexington, KY, United States; ^3^Department of Psychiatry and Behavioral Sciences, University of California, San Francisco, San Francisco, CA, United States; ^4^Department of Psychology, Yale University, New Haven, CT, United States

**Keywords:** sleep/wake regularity, sleep, executive function, stress, cognition

## Abstract

**Introduction:**

Sleep and stress processes shape executive function. Evidence suggests that poor sleep regulation can lead to significant impairments in executive functions. Psychological stress can also directly impact a variety of executive functions, often leading to declines, but may additionally reduce executive function via its negative impact on sleep. Rates of perceived stress and poor sleep have skyrocketed in recent years. As such, it has become increasingly important to understand how daily stress exposures and sleep processes modulate executive functions.

**Methods:**

In a remote 21-day app-based study, 227 participants completed sleep and stress assessments three times a day. They also completed three executive functioning tasks at various timepoints across the 21-day study interval that assessed cognitive inhibition (Emotional Stroop task), cognitive flexibility (Trail Making Test A and B), and working memory (Backwards Digit Span).

**Results:**

Participants with consistent sleep/wake schedules reported fewer acute stress events when compared to those with inconsistent sleep schedules. Those with greater sleep/wake regularity also had faster responses to self-relevant negative probes (vs. general negative and neutral) in the Stroop task. Further, variability in sleep/wake timing and reported acute stress exposures across the 21-day study interval interacted to predict performance on the Emotional Stroop task. Specifically, as the number of acute stress events experienced across the 21-day interval increased, participants with more regular sleep schedules had slower overall response times on the Stroop. Higher acute stress exposures led to specific response time delays to neutral and self-relevant negative probes for those with high sleep/wake regularity. We found no impact of the number of acute stress events or stress intensities on working memory span, Stroop accuracy, or Trails response time.

**Discussion:**

These data may indicate that sleep/wake regularity preserves adaptive inhibitory control responses to cumulative acute stress.

## Introduction

Sleep and stress are each complex biobehavioral processes that shape daily psychosocial interactions partially through their impact on executive functions. Specifically, short sleep durations (Tai et al., 2022), poor self-reported sleep quality (Rana et al., 2018), and variability in sleep/wake timing (Kuula et al., 2017) can hinder executive functions. This has been evidenced in studies showing that individuals with short sleep periods, low sleep quality, and irregular sleep schedules exhibit poorer working memory and slower attentional and set-shifting abilities. In addition, psychological stress exposures often lead to significant sleep loss (Kim and Dimsdale, 2007; Slavish et al., 2021) and can independently hinder executive functions (Wang et al., 2019; Kan et al., 2021). Empirical studies that detail how stress exposures and sleep together determine daily executive function are limited in number. With more than 80% of adults reporting high levels of psychological distress in the U.S. (Bethune, 2021), 33% reporting not getting enough sleep, and 40% reporting unintentionally falling asleep during daytime hours at least once a month (National Institute of Health., 2022), understanding how daily stress and sleep interact to impact executive functions will better contextualize the costs of these compounding social realities and provide insight into how to mitigate these growing public health concerns. Here, we explored how indicators of sleep, sleep/wake regularity, and daily stress exert their individual and interactive effects on three executive functions—cognitive inhibition, flexibility, and working memory—in a daily diary and cognitive behavior study.

Executive functions are defined by higher-order cognitions and can be divided into organizational and regulatory processes. Examples of organizational functions include attention, working memory, cognitive flexibility, and planning, whereas examples of regulatory functions include initiation or inhibition of cognition or behavior, self-control, and emotion regulation (Baggetta and Alexander, 2016). Research studies have demonstrated that executive function ability peaks and dips across the 24-h day and that alignment between an individual's internal psychophysiological resources and environmental demands (i.e., entrainment) can benefit executive function (Manly et al., 2002; Wright et al., 2002; Horowitz et al., 2003), whereas dysregulation or misalignment can lead to impairments (Gritton et al., 2012; Gruber and Cassoff, 2014). For example, studies indicate that greater variability in sleep/wake timing may negatively impact inhibitory function, working memory, and set-shifting aspects of flexibility (Sagaspe et al., 2012; Cheng et al., 2017; Valdez, 2019; Taillard et al., 2021); however other studies suggest that certain executive functions, such as attention, may not be as sensitive to sleep/wake regularity (Bratzke et al., 2012; Collet et al., 2020). Executive functions also vary in their vulnerability to sleep loss with cognitive inhibition, working memory, and cognitive flexibility being more sensitive to sleep dysfunction. However, attentional processes remain more resilient (Nilsson et al., 2005; Frenda and Fenn, 2016; Honn et al., 2019; Skurvydas et al., 2019). For example, after controlling for relevant lapses in attention that often follow sleep deprivation, inhibitory control deficits still remain (Mao et al., 2021).

Sleep-related loss to executive functions may also come at a consequence to affective processing. For example, alterations in affective regulation due to sleep-dependent loss in cognitive inhibition have been noted. Explicitly, after sleep loss, inhibitory responses are often biased toward negative stimuli (Killgore, 2010; Tempesta et al., 2010, 2018; Watling et al., 2017). In one study, after 32 participants underwent 36 h of total sleep deprivation, they failed to inhibit responses to negative vs. positive stimuli on an affective Go/NoGo task, which was demonstrated by participants responding faster to stimuli with negative vs. positive valences (Anderson and Platten, 2011). Additionally, poor self-reported sleep quality has been linked to increased reactivity to information with negative valence (Fairholme and Manber, 2015), however similar valence-dependent response patterns have not been observed in non-sleep deprived controls (Anderson and Platten, 2011). Given this pattern of results, it has been proposed that executive functions, specifically cognitive inhibition, may be moderated by affect because sleep loss enhances the attentional bias toward negative stimuli. This pattern of cognitive/affective interactions thus enhances the likelihood that sleep deficits will result in detectable changes in behavioral responses to information with negative valence (Lee et al., 2022). This sleep-affect interaction is not present in all executive functions. Studies exploring affective manipulations of working memory have shown no difference in accuracy or response time for negative or positive valence items on N-back tasks between sleep deprived and non-sleep deprived individuals (Tempesta et al., 2014; Gerhardsson et al., 2018). The same holds true for cognitive flexibility, where while the impact of sleep deprivation on flexibility has not been directly measured, studies comparing patients with mood disorders to those without diagnoses have shown consistent cognitive flexibility deficits following poor sleep in both those with and without clinical diagnoses. Together, this set of data suggests that cognitive inhibition may be one executive function uniquely sensitive to sleep loss. In this study, we utilized an inhibition task with affective manipulations to shed further light on these relationships.

Daily psychological stress is a key factor that impacts sleep duration, quality, and sleep/wake timing. Ample evidence suggests that psychological stress exposures often lead to sleep loss by increasing sleep onset latencies and decreasing total sleep durations and sleep quality (Heslop et al., 2002; Kim and Dimsdale, 2007). Additionally, in one study with 80 participants who underwent 7 days of stress and sleep assessment with sleep diaries, actigraphy, and EEG, researchers found a bi-directional relationship between stress and sleep. On days when participants reported experiencing an acute stressor, they also reported decreased sleep durations. Greater wake after sleep onset (WASO) was also associated with greater severity of next-day stress (Slavish et al., 2021). In a separate study, 552 participants completed 56 days of sleep, stress, and affect diaries. Here, high levels of sleep quality and positive affect combined to protect participants against stress-induced increases in negative affect (Blaxton et al., 2017). Taken together, these results suggest that acute stressors and sleep can bidirectionally influence one another and that affect may be an important moderator—with negative affect exacerbating and positive affect mitigating—the reciprocal process of sleep loss and stress.

Psychological stress often hinders cognitive flexibility, inhibition, and working memory as neural and cognitive resources are directed toward other priorities including (Sandi, 2013) vigilance, attention, and acquisition of stress-relevant information (Shields et al., 2016; Degroote et al., 2020). For example, one study found that participants randomized to a stress induction vs. those in a no-stress control group were less accurate and slower to respond on a cognitive inhibition task (Starcke et al., 2016). Yet, in a different study, acute stress boosted attentional ability (Shields et al., 2019). In this study, participants had faster response times with intact accuracy on selective attention tasks after being exposed to an acute stress manipulation compared to a control group (Shields et al., 2019). While the results of these two studies are contradictory, it is important to note key differences between these projects including (1) different assessments of cognition, (2) different age demographics, and (3) psychological (Starcke et al., 2016) vs. physiological (Shields et al., 2019) stress induction tasks. These key differences likely impacted the disparate findings between these two examinations and highlight the need for extended study.

Importantly, the individual effects that sleep and stress exert on executive functions are at least partially dependent on the effects that they each exert on the other. For example, studies examining the combined influences of sleep and stress on cognition have found that shorter sleep durations lead to reduced attentional processing when physiological stress is high (Thompson et al., 2022), and that working memory improvements following acute stress are moderated by sleep quality (Eskildsen et al., 2017). Critically, the studies reviewed herein have only examined either a snapshot of behavior in a laboratory setting or reports of sleep and stress paired with a single assessment of cognitive ability. These study designs have resulted in gaps regarding how daily stress and sleep processes transpire across several days in real world settings to impact executive functions.

The aim of this study was to examine how indicators of sleep and stress work together to influence daily executive functions. This study was conducted via a mobile application in an international population where participants completed mobile-based cognitive tasks and self-reported sleep and stress. We hypothesized that working memory, cognitive inhibition, and cognitive flexibility would be sensitive to sleep factors and daily stressors. We expected that the influence of daily stress on executive function would be moderated by sleep duration, quality, and sleep/wake regularity, such that when people experienced an acute stressor but also had longer sleep durations, better sleep quality, and more regular sleep/wake patterns they would demonstrate better working memory, greater flexibility, and greater cognitive inhibition, specifically for negative stimuli.

## Methods

### Participants

Participants self-selected into the study by downloading an app called *MyBPLab* that was available on the Google PlayStore and was designed as a 21-day study that included three prompts a day and focused on stress and emotion in daily life. Enrolling in the study allowed people to access a specialized optic sensor that measured blood pressure (see Gordon and Mendes, 2021). This sensor was available for select Samsung phones and watches, and individuals who owned these devices could seek out the app on the PlayStore or the Samsung website and request to join the study. No active recruitment efforts were used. For this analysis, we focus on 227 participants (male = 163, female = 64; mean age = 52.51, SD = 11.50, min = 18, max = 80; non-US based = 74) who had sufficient data based on our inclusion criteria, which was defined by completion of at least one of the three cognitive tasks, 21 days of sleep data, and self-report questionnaires. Participants were included if they passed an English fluency test after downloading the app, were at least 18 years old, completed all 21 days of the study, which included all 21 sleep diaries, and at least one of the cognitive tasks. Participants self-reported their race and ethnicity: 92% non-Latino and 8% Latino; 76% White; 8% Asian/Pacific Islander, 6% Black, 3.5% Indian, 2% Native American, and 3.5% declined to report.

### Study protocol

After downloading the app and confirming inclusion criteria, participants completed the consent form, provided demographic information (race, ethnicity, age, sex, education, country location, and health information) and received authorization to participate in the study via email. Once enrolled, participants were asked to access the app three times per day, occurring at random times in the morning (between 06:00 and 10:00), afternoon (between 12:00 and 15:00), and evening (between 18:00 and 22:00) to complete daily diaries and cognitive tasks. Each cognitive assessment was presented to participants every 3–5 days. Each task could only be completed once per day, and to minimize circadian effects on cognitive task performance, tasks were staggered across the duration of the study depending on which timeframe (morning, afternoon, or evening) that the tasks first occurred. For example, if the first instance the participant received a task was on the evening of Day 2, then throughout the remainder of the study, they would only receive that task during subsequent evening sessions. Tasks were not compulsory, and participants could opt out of completion of any task throughout the duration of the study. Prior to completing each task, participants were provided detailed instructions and a short practice session to ensure understanding.

### Cognitive assessments

#### Emotional Stroop task

**Figure 1:**
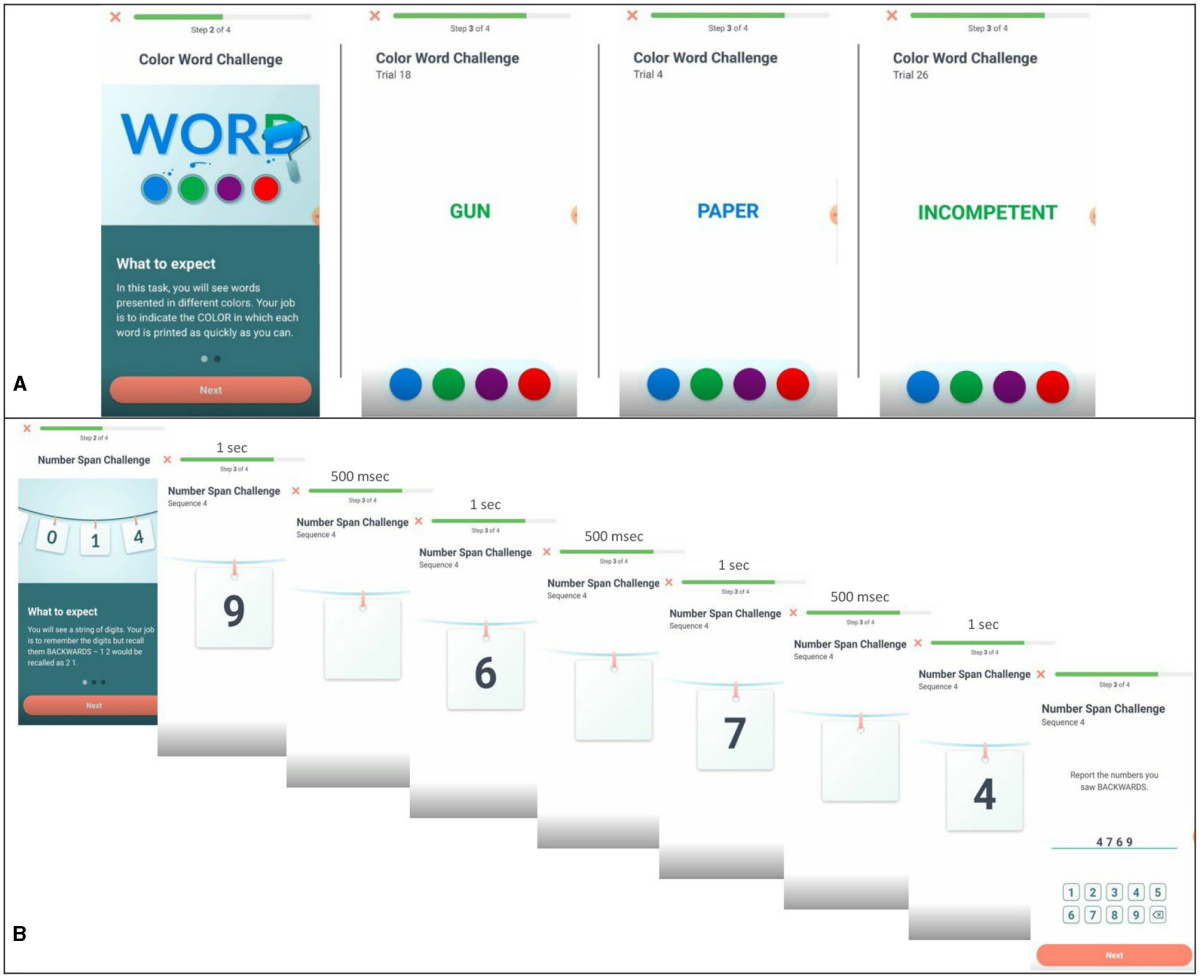
Examples of task presentation for Emotional Stroop and Backwards Digit Span. **(A)** Emotional Stroop task displaying negative, neutral, and self-relevant negative word types. **(B)** Backwards digit-span task for a four-digit sequence.

This task is a measure of affective regulation and cognitive inhibition (Ben-Haim et al., 2016). Participants were provided the opportunity to complete this task during afternoon sessions on study days 1, 5, 10, 14, and 19. Participants were presented with a series of words in different colors (blue, red, green, and violet) at the center of their screen and asked to identify the color of the word as quickly and accurately as possible while ignoring the meaning of the word. Three different word types: neutral (e.g., couch), negative (e.g., bomb), and self-relevant negative (e.g., inferior) were presented to participants in random order (see Figure 1A). See Supplementary Table 1 for word lists. The task consisted of 30 trials (10 neutral, 10 negative, and 10 self-relevant negative) and participants were initially given a 1,250 ms time interval to make their response which was increased by 200 ms following incorrect responses and decreased by 100 ms following correct responses in an effort to mitigate some aspects of task-dependent learning. Color-word assignment was pseudo random and designed to equally represent each of the four colors within each of the three categories across the five testing sessions of the Stroop. However, as the task was not compulsory, and due to the limited sample we present here, color-word assignment resulted in the following combinations of word-types and colors: Neutral: Blue (28.8%), Green (29.7%), Red (19.2%), Violet (22.4%); Negative: Blue (22.9%), Green (25.5%), Red (27.5%), Violet (24.0%); Self-relevant negative: Blue (26.4%), Green (37.7%), Red (16.1%), Violet (19.7%). Outcomes of interest for this task are accuracy (responding with the correct color of each word) and response time for correct trials (the time between stimulus presentation and participant response in milliseconds) for each word type.

#### Backwards Digit Span

This task is a standard measure of working memory span (Woods et al., 2015). Participants were presented with a sequence of numbers (ranging in length from 3 to 9 digits) and asked to type the sequence they were presented in reverse order. Each digit was displayed on the center of the screen for 1 s, separated by a 500 ms blank screen between each presented digit (see Figure 1B). For example, if participants were presented “6925,” the correct response would be “5296.” All participants received up to two opportunities to correctly report a reversed sequence of each sequence length. If successful, participants were presented with sequences of increasing length. For example, if they correctly reversed a sequence of three digits, they moved on to a sequence of four digits, then five digits, and so on. If unsuccessful after two attempts, the task ends. Each task consisted of up to 12 number sequences. Outcomes of interest for this task are the length of the longest sequence correctly completed and response time. Participants saw this task in the morning on study days 3, 4, 9, 12, 13, 18, and 21.

#### Trail Making Test A and B

**Figure 2:**
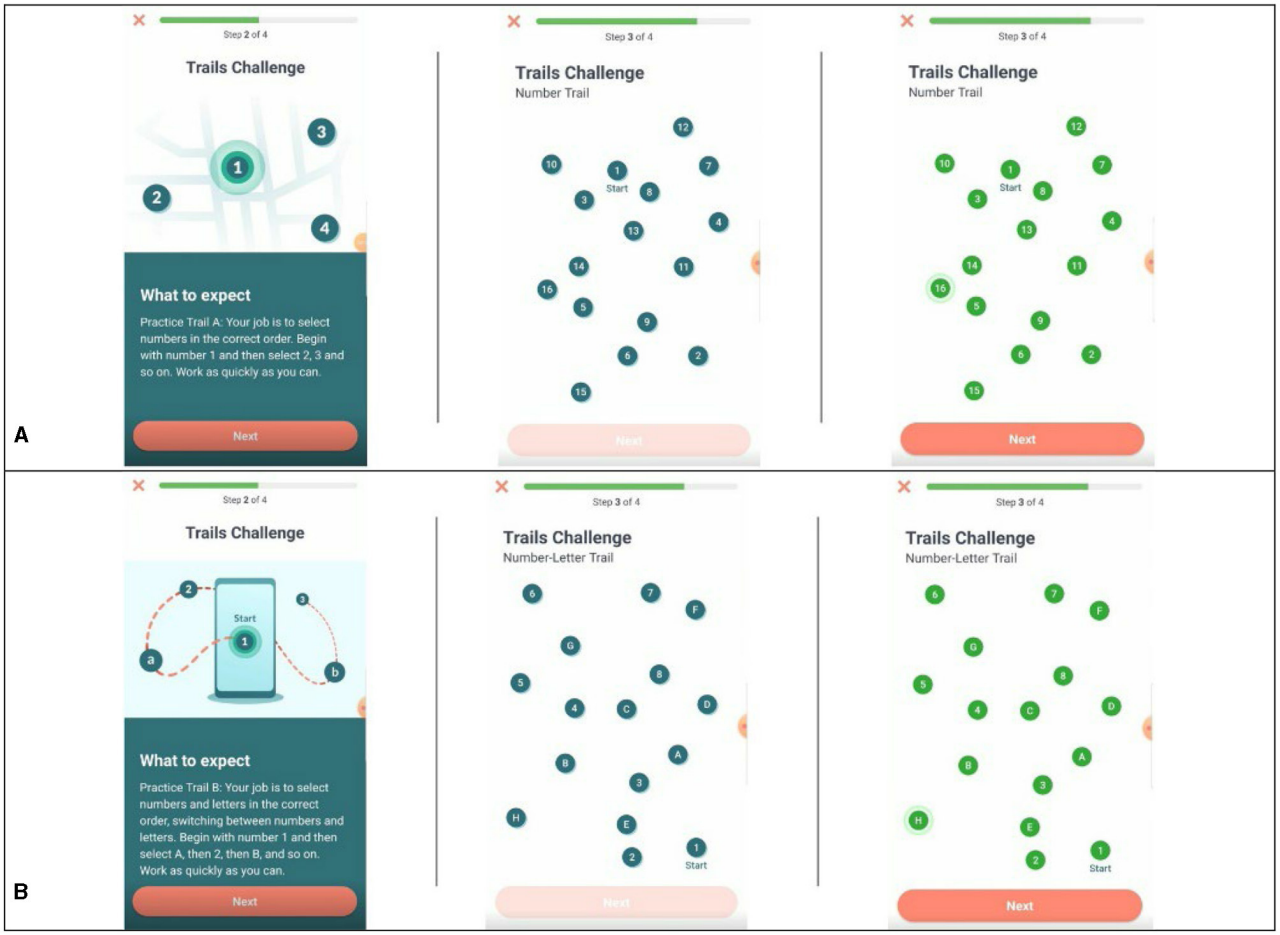
Examples of task presentation for Trail Making Test A and B. **(A)** Example of Trail A pre- and post-completion. **(B)** Example of Trail B pre- and post-completion.

This task comprises two parts, Trail A and Trail B, that evaluate aspects of cognitive flexibility (Bowie and Harvey, 2006). In Trail A, participants were presented with 25 circles labeled #1–25 and randomly distributed on the screen. Participants were cued to the start point, #1, and asked to identify and draw a line between numbers in ascending order as quickly as possible using their finger. In Trail B, participants were again presented 25 circles, half were labeled with numbers, #1–13, and the other half were labeled with letters, A–L. Participants were asked to identify and draw a line in ascending order alternating between numbers and letters (e.g., 1-A-2-B-3-C-4-D) as quickly as possible (see Figures 2A, B). Outcomes of interest include the time to complete each trail and the difference in completion time between Trail A and Trail B. Participants saw this task in the afternoon on days 3, 7, 9, 12, 16, 18, and 21.

#### Distraction probes

At the end of each task, participants were asked to report on two questions: “How much of the time during the last task were you engaged and totally focused on what you were doing?” and “Were you significantly interrupted while you were completing the task?” on a scale of 1 “not at all” to 5 “a great deal.” These probes were used to assess participant-reported distraction given tests were not conducted in a controlled laboratory setting.

### Daily surveys

#### Sleep/wake timing

Participants were provided with a questionnaire modified from the Pittsburgh Sleep Quality Index (Buysse et al., 1989) at each morning session, and asked to report what time of day they went to sleep the night prior and what time they woke up the next morning. Participants also reported the number of hours they slept the night prior with a scroll bar from 0 to 12+ h. This was used as our measure of daily total sleep time (TST). How long it took each participant to fall asleep was also reported in minutes and this was used as a measure of sleep onset latency (SOL). Participants rated the quality of their sleep on a scale of 1 “very bad” to 4 “very good” and we utilized this value as our measure of sleep quality. TST, SOL, and sleep quality were included as day-of task predictors in all models.

To assess sleep regularity, we calculated the mid-point of participants' sleep periods from their self-reported bed and wake times for each of the 21 days. For example, if a participant reported a bedtime of 11:00 p.m. and a wake time of 7:00 a.m., then their mid-sleep point would be 4:00 a.m. We then calculated the sum of the sequential difference scores between the 21 mid-points and took the root mean square to create a sleep regularity score for each participant, which was representative of sequential consistency or inconsistency in sleep/wake schedule with lower scores representing more consistent sleep schedules.

#### Stress

At each check-in, participants were presented with a modified scale from the Challenge and Threat Appraisal Questionnaire (Mendes et al., 2007). They were asked “Have you experienced any particularly stressful events since your last check-in?” The total count of participants who reported “yes” responses was calculated across all check-ins and served as a measure of acute stress exposures. If they answered “yes,” they were then asked to report how stressful the event was on a scale of 1 “not at all” to 5 “extremely.” If participants answered “no,” they were then asked to report if they “currently felt stressed, anxious, or overwhelmed.” Participants answered on a scale of 1 “not at all” to 5 “extremely.” This prompt was modified from the Perceived Stress Scale (Cohen et al., 1983). For our purposes, we conceptualized these assessments as indicators of momentary stress and included them in each model as a measure of daily stress intensity (not averaged across the 21-days).

### Health and education factors

Overall health was assessed with responses to the question “In general, would you say your health is:” to which participants were presented with the scale 1 “Poor” to 5 “Excellent.” Physical exercise was assessed with the Yes/No question “Do you exercise regularly (defined as more than 3 × a week)?” The question “What is the highest level of education that you completed?” evaluated education level and was answered on a scale of 1 “No high school diploma” to 6 “Graduate school degree.”

### Statistical approach

Pearson correlation coefficients were calculated to examine bivariate relationships between sleep, stress, and cognitive outcomes. To further characterize participants based on their sleep regularity and better understand potential interactions, participants were added to one of three groups. Group 1 was “high regularity” where participants' sleep regularity scores fell in the top 10% of the score distribution, Group 2 contained participants in the middle 80% of the distribution, and Group 3 was “low regularity” where scores fell in the bottom 10% of the distribution. This grouping variable was used for two purposes: (1) to determine how stress was stratified across these three sleep regularity groups and (2) for visualization purposes. Linear mixed effect models were used (estimated using REML with participants as random effects) to assess the impact of sleep and stress factors on cognitive performance given their longitudinal and repeated structure. The natural log of the sleep regularity score was taken to normalize their distribution and entered as a continuous variable in all models. When applicable, between-person variables, including sleep regularity, were grand mean centered and within-person variables were cluster centered.

## Results

**Figure 3:**
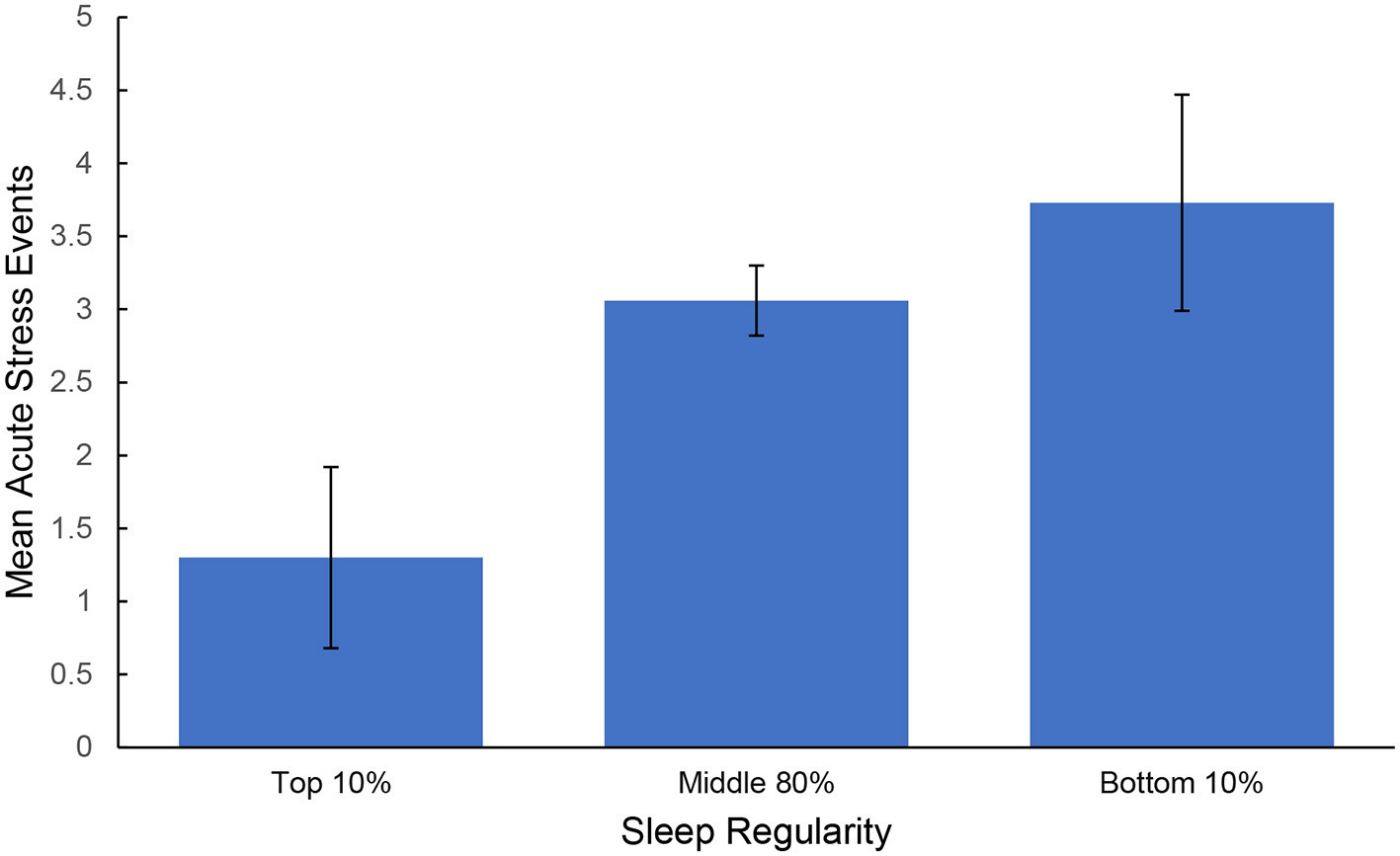
Average count of acute stress events experienced by sleep regularity groups. Error bars represent standard error of the mean.

The two distraction probe questions were assessed to determine participant engagement. Overall, participants reported high levels of engagement with the tasks [Stroop: *M* = 4.496, SD = 0.843; Trail Making Test A and B (TMT): *M* = 4.039, SD = 1.143; Digit: *M* = 3.733, SD = 1.183] and minimal interruptions (Stroop: 1.386, SD = 0.812; TMT: *M* = 1.673, SD = 1.053; Digit: *M* = 2.118, SD = 1.276). Table 1 includes bivariate correlations between average sleep, stress, and the cognitive assessments across individuals. Stronger sleep regularity correlated with longer TST (*r* = −0.198, *p* = 0.003), shorter SOL (*r* = 0.230, *p* < 0.001), greater sleep quality (*r* = −0.163, *p* = 0.014), and fewer reported acute stress events (*r* = 0.154, *p* = 0.021). Larger counts of acute stress events were correlated with lower sleep quality (*r* = −0.138, *p* = 0.038), and greater stress intensity was correlated with lower TST (*r* = −0.175, *p* = 0.008), sleep quality (*r* = −0.273, *p* < 0.001), and longer SOL (*r* = 0.203, *p* = 0.002). A one-way ANOVA was conducted to assess any differences in the number of acute stress exposures experienced by sleep regularity groups. The overall model was significant [*F*_(2, 224)_ = 3.380, *p* = 0.036, η^2^ = 0.030] and Dunn-Bonferroni *post-hoc* comparisons revealed that individuals with low sleep/wake regularity experienced more acute stress events (*M* = 3.727, SD = 3.467) than the high sleep/wake regularity group (*M* = 1.300, SD = 2.755), *p* = 0.046. No significant differences were found between comparisons with the medium group (*M* = 3.060, SD = 3.231). See Figure 3. No differences were found in reported stress intensity [*F*_(2, 224)_ = 1.850, *p* = 0.159].

**Table 1:** Correlations between sleep, stress, and cognitive assessments.

	**TST**	**Sleep quality**	**SOL**	**Sleep Reg**.	**Acute stress**	**Stress Inten**.	**Stroop RT**	**Stroop Acc**.	**Trail A**	**Trail B**	**Trail B-A**
TST	–										
Sleep quality	0.178^**^	–									
SOL	−0.375^***^	−0.411^***^	–								
Sleep Reg.	−0.198^**^	−0.163^*^	0.230^***^	–							
Acute stress	−0.052	−0.138^*^	−0.066	0.154^*^	–						
Stress inten.	−0.175^**^	−0.273^***^	0.203^**^	0.100	0.089	–					
Stroop RT	−0.051	−0.014	0.033	0.018	0.030	−0.164^*^	–				
Stroop Acc.	−0.022	0.075	0.031	−0.087	0.049	0.107	−0.317^***^	–			
Trail A	0.098	0.120	−0.174	0.051	0.169	−0.094	0.502^***^	−0.046	–		
Trail B	0.068	−0.085	−0.013	−0.195	−0.009	0.024	0.357^**^	0.021	0.648^***^	–	
Trail B-A	0.059	−0.054	0.001	−0.139	−0.149	−0.019	−0.080	0.129	0.019	0.524^***^	–
Digit length	0.058	0.130	−0.267^***^	0.011	−0.024	−0.132	−0.274^***^	0.051	−0.119	−0.299^**^	−0.281

^*^p <0.05.

^**^p <0.01.

^***^p <0.001.

TST, total sleep time; SOL, sleep onset latency; Sleep Reg, sleep regularity; Stress Inten, stress intensity; Stroop RT, Stroop reaction time; Stroop Acc, Stroop accuracy.

A one-way ANOVA was also conducted to assess differences in TST, SOL, and sleep quality between the sleep regularity groups. The overall models were not significant for TST [*F*_(2, 224)_ = 2.800, *p* = 0.063, η^2^ = 0.020] or sleep quality [*F*_(2, 224)_ = 1.700, *p* = 0.184, η^2^ = 0.010], indicating no statistical difference in TST or sleep quality between the high sleep regularity (TST: *M* = 7.271, SD = 0.807; Sleep Quality: *M* = 2.945, SD = 0.379) and low sleep regularity groups (TST: *M* = 6.665, SD = 1.230; Sleep Quality: *M* = 2.781, SD = 0.334). For SOL, the overall model was significant [*F*_(2, 223)_ = 3.330, *p* = 0.038, η^2^ = 0.030] and Dunn-Bonferroni *post-hoc* comparisons revealed that individuals with low sleep/wake regularity reported longer SOL (*M* = 28.042, SD = 20.271) than the medium group (*M* = 18.487, SD = 16.610). No significant differences were found between comparisons with the high regularity group (*M* = 18.614, SD = 8.823).

### Emotional stroop

**Figure 4:**
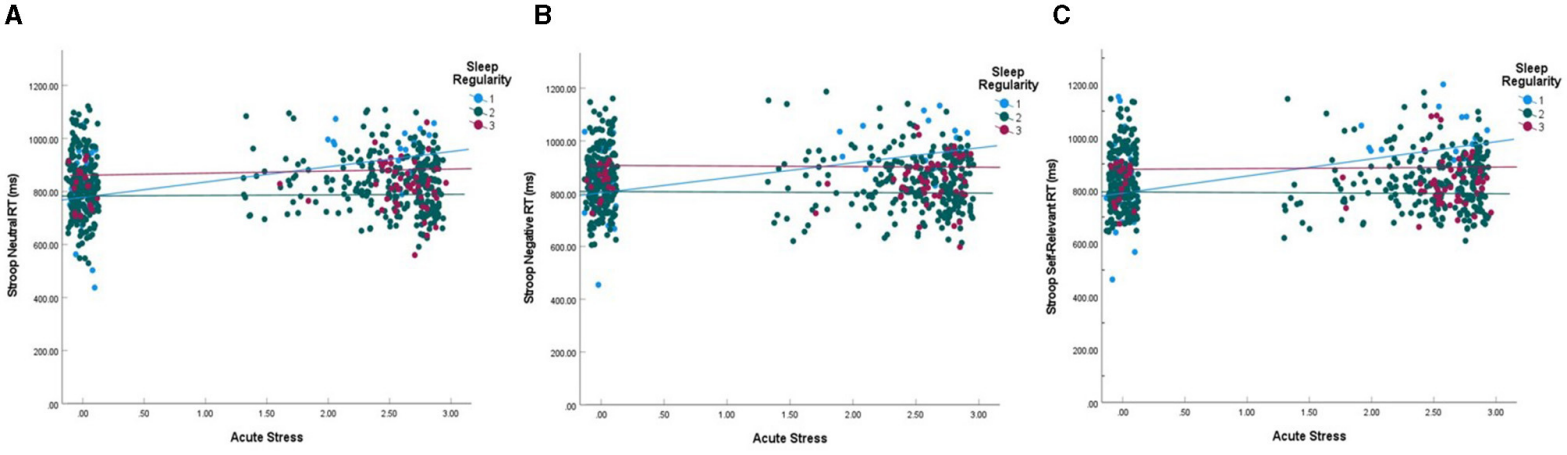
Interaction between acute stress event counts and sleep regularity on stroop RT. **(A)** Response time for neutral words. **(B)** Response time for negative words. **(C)** Response time for self-relevant negative words. Interactions were statistically significant for neutral (*p* = 0.024) and self-relevant negative words (*p* = 0.046). Blue lines represent the top 10% of sleep regularity (group 1), red represent the lowest 10% (group 3), and green represent the middle 80% (group 2). Acute stress is RMS transformed and data points are jittered vertically to enhance visualization.

One hundred sixty-eight participants (Max *N*_observations_ = 566) completed the Emotional Stroop task. Performance on this task was analyzed within each word type as well as averaged across all word types (overall performance). Greater stress intensity was correlated with faster overall response times (*r* = −0.164, *p* = 0.033). Correlations within word type also emerged with faster response times to negative (*r* = −0.166, *p* = 0.031) and self-relevant negative words (*r* = −0.179, *p* = 0.020) associated with lower self-reported stress intensities. Tables 2, 3 include all model results for Emotional Stroop performance by word type. For overall performance, age was a significant predictor where younger participants demonstrated faster response times (*b* = 4.392, *p* < 0.001). Age also predicted greater accuracy for overall performance (*b* = −0.005, *p* < 0.001), again with younger participants performing better. Age was also related to response time for neutral (*b* = 4.072, *p* < 0.001), negative (*b* = 4.529, *p* < 0.001), and self-relevant negative words (*b* = 4.577, *p* < 0.001) as well as accuracy and for neutral (*b* = −0.004, *p* < 0.001), negative (*b* = −0.003, *p* = 0.002) and self-relevant negative words (*b* = −0.003, *p* < 0.001). As expected, participants displaying more regular sleep/wake schedules had faster overall response times on the Stroop task (*b* = 20.981, *p* = 0.049) and specifically to self-relevant negative words (*b* = 23.857, *p* = 0.047). Participants who reported more acute stress events duringthe study were slower to respond overall on the task (*b* = 60.869, *p* = 0.012) and specificallyto neutral (*b* = 65.344, *p* = 0.016) and self-relevant negative words (*b* = 52.162, *p* = 0.046), indicating poorer inhibition. Significant interactions between acute stress exposures and sleep regularity also emergedfor overall performance (*b* = −2.832, *p* = 0.012) in addition to neutral (*b* = −2.924, *p* = 0.024) and self-relevant negative words (*b* = −2.500, *p* = 0.046), indicating that on days when participants reported a stress exposure, those with higher regularity were slower to respond on the Stroop (see Figure 4). For self-relevant negative words, participants located in the U.S. were slower to respond (*b* = 28.499, *p* = 0.027). No difference in accuracy was found between the high and low regularity groups for overall performance or neutral and negative word types.

**Table 2:** Linear mixed model results for emotional stroop reaction time by word type.

	**Neutral**	**Negative**	**Self-relevant**
(Intercept)	−229.010 (−5.130)^***^	−223.220 (−5.150)^***^	−229.950 (5.320)^***^
Total sleep time	−0.195 (−0.060)	2.437 (0.820)	0.921 (0.320)
Sleep quality	−7.660 (−1.190)	−2.014 (−0.340)	0.882 (0.160)
Sleep onset latency	−0.158 (−0.730)	0.046 (0.230)	−0.022 (−0.120)
Sleep regularity	14.364 (1.120)	8.344 (0.680)	23.857 (2.000)^*^
Acute stress	65.344 (2.440)^*^	37.422 (1.430)	52.162 (2.010)^*^
Stress intensity	−23.267 (−0.510)	−50.128 (−1.190)	17.033 (0.420)
Acute × regularity	−2.924 (−2.280)^*^	−1.844 (−1.470)	−2.500 (−2.010)^*^
Intensity × regularity	2.142 (0.370)	5.457 (1.020)	−3.381 (−0.670)
Sex	20.307 (1.440)	7.040 (0.510)	−2.702 (−0.200)
Age	4.072 (7.290)^***^	4.529 (8.310)^***^	4.577 (8.420)^***^
Exercise	−14.630 (−1.100)	−14.582 (−1.130)	−20.383 (−1.580)
Ethnicity	−39.015 (−1.190)	−25.917 (−0.810)	−32.565 (−1.020)
Education	0.183 (0.040)	1.109 (0.260)	−0.480 (−0.110)
Health	−10.560 (−1.300)	−8.176 (−1.030)	−10.008 (−1.260)
Country	19.613 (1.500)	9.356 (0.730)	28.499 (2.240)^*^
Random effect	4,235.550 (6.250)^***^	4,211.61 (6.670)^***^	4,215.900 (6.730)^***^
AIC	6,450.100	6,291.000	6,265.800

^*^p <0.05.

^***^p <0.001.

Statistics reported are B (t-value).

**Table 3:** Linear mixed model results for emotional stroop accuracy by word type.

	**Neutral**	**Negative**	**Self-relevant**
(Intercept)	1.161 (19.320)^***^	1.076 (16.180)^***^	1.139 (20.450)^***^
Total sleep time	−0.004 (−0.710)	−0.001 (−0.180)	−0.002 (−0.360)
Sleep quality	−0.002 (−0.180)	0.001 (0.110)	0.011 (1.360)
Sleep onset latency	0.001 (1.390)	−0.000 (−0.490)	0.000 (0.690)
Sleep regularity	0.023 (1.190)	−0.003 (−0.160)	−0.005 (−0.330)
Acute stress	0.009 (0.260)	0.020 (0.510)	−0.005 (−0.140)
Stress intensity	0.156 (1.950)	0.011 (0.170)	−0.015 (−0.260)
Acute × regularity	−0.001 (−0.300)	−0.001 (−0.410)	0.000 (0.260)
Intensity × regularity	−0.019 (−1.850)	0.000 (0.030)	0.003 (0.420)
Sex	0.009 (0.460)	0.010 (0.490)	0.012 (0.700)
Age	−0.004 (−4.670)^***^	−0.003 (−3.130)^**^	−0.003 (−4.450)^***^
Exercise	0.006 (0.320)	−0.005 (−0.270)	0.010 (0.590)
Ethnicity	0.007 (0.170)	0.010 (0.210)	0.011 (0.260)
Education	−0.004 (−0.600)	−0.005 (−0.820)	−0.008 (−1.520)
Health	−0.013 (−1.180)	−0.002 (−0.160)	−0.012 (−1.220)
Country	−0.043 (−2.480)^*^	−0.013 (−0.640)	−0.017 (−1.020)
Random effect	0.005 (4.140)^***^	0.010 (6.680)^***^	0.007 (6.300)^***^
AIC	−509.900	−762.200	−851.400

^*^p <0.05.

^**^p <0.01.

^***^p <0.001.

Statistics reported are B (t-value).

### Trail Making Test

One hundred participants (Max *N*_observations_ = 198) completed the Trail Making Test. Table 4 includes all model results for Trail Making performance. Age was a significant predictor where younger participants demonstrated faster completion times for Trail A (*b* = 190.470, *p* < 0.001) and Trail B (*b* = 302.210, *p* < 0.001). No other significant effects were found for either trail or the difference between completion time for Trail B and Trail A.

**Table 4:** Linear mixed model results for trail making task completion times.

	**Trail A**	**Trail B**	**Trail B–Trail A**
(Intercept)	−12,855.000 (−3.580)^***^	−18,802.000 (−2.840)^**^	4,288.520 (0.520)
Total sleep time	−157.530 (−0.480)	449.950 (1.020)	98.296 (0.170)
Sleep quality	−286.130 (−0.500)	400.430 (0.500)	1,353.430 (1.430)
Sleep onset latency	−2.501 (−0.120)	10.293 (0.330)	−12.637 (−0.430)
Sleep regularity	802.080 (0.530)	1,884.000 (0.770)	4,687.130 (1.310)
Acute stress	−3,524.920 (−1.490)	2,273.350 (0.580)	1,689.070 (0.300)
Stress intensity	3,491.590 (0.550)	17,034.000 (1.510)	22,316.000 (1.730)
Acute × regularity	209.450 (1.820)	−85.423 (−0.450)	−89.512 (−0.320)
Intensity × regularity	−476.550 (−0.570)	−2,158.100 (−1.480)	−3,181.390 (−1.880)
Sex	714.210 (0.700)	3,172.800 (1.660)	−229.520 (−0.110)
Age	190.470 (4.430)^***^	302.210 (3.680)^***^	76.944 (0.760)
Exercise	142.880 (0.140)	257.880 (0.140)	−1,048.440 (−0.530)
Ethnicity	266.910 (0.120)	1,561.430 (0.420)	−7,376.940 (−0.770)
Education	119.060 (0.320)	29.407 (0.040)	270.320 (0.330)
Health	−54.870 (−0.090)	−769.840 (−0.670)	−1,740.580 (−1.640)
Country	1,117.090 (1.110)	198.310 (0.100)	−1,148.580 (−0.570)
Random effect	16,082,138.000 (4.860)^***^	58,386,983.000 (5.140)^***^	22,405,593.000 (2.810)^**^
AIC	3,132.300	3,827.700	1,210.200

^**^p <0.01.

^***^p <0.001.

Statistics reported are B (t-value).

### Digit span

One hundred sixty-three participants (Max *N*_observations_ = 592) completed the Digit Span. Longer SOL was correlated with shorter sequence length completion (*r* = −0.267, *p* < 0.001). Table 5 includes all model results for Digit Span performance. Overall health predicted completion of longer sequences on the digit span task (*b* = 0.356, *p* = 0.007). Younger age (*b* = 0.001, *p* = 0.026) predicted faster completion of the digit sequences. No other significant effects were found.

**Table 5:** Linear mixed model results for digit span.

	**String length**	**Response time**
(Intercept)	−0.513 (−0.700)	−0.096 (−2.640)^**^
Total sleep time	0.015 (0.360)	0.001 (0.160)
Sleep quality	0.035 (0.410)	−0.003 (−0.400)
Sleep onset latency	0.002 (0.650)	0.000 (0.430)
Sleep regularity	0.157 (0.670)	0.018 (1.310)
Acute stress	−0.100 (−0.220)	−0.023 (−0.980)
Stress intensity	0.408 (0.460)	0.117 (1.860)
Acute × regularity	0.003 (0.130)	0.001 (0.970)
Intensity × regularity	−0.061 (−0.540)	−0.014 (−1.670)
Sex	0.247 (1.040)	0.009 (0.830)
Age	−0.015 (−1.610)	0.001 (2.25)^*^
Exercise	−0.021 (−0.100)	−0.005 (−0.450)
Ethnicity	−0.279 (−0.570)	−0.008 (−0.350)
Education	−0.013 (−0.190)	−0.003 (−0.730)
Health	0.356 (2.730)^**^	0.004 (0.620)
Country	0.302 (1.380)	0.014 (1.350)
Random effect	1.298 (6.820)^***^	0.001 (2.500)^**^
AIC	1,902.100	−909.300

^*^p <0.05.

^**^p <0.01.

^***^p <0.001.

Statistics reported are B (t-value).

## Discussion

The aim of this study was to examine the daily relationship between indicators of sleep and stress, and how they exert independent and cumulative effects on cognitive inhibition, working memory, and flexibility. To this end, we assessed performance on an Emotional Stroop, Trail Making Test, and Backwards Digit Span using subjective reports of total sleep time, sleep quality, sleep/wake regularity, and stress collected via a mobile application. As expected, participants with more regular sleep schedules displayed better inhibition, specifically for self-relevant negative stimuli.

While we hypothesized that sleep regularity would moderate the relationship between stress and executive functioning, this was only the case for inhibition. Here, we found that for individuals with consistent sleep/wake rhythms, as stress exposures increased across the 21-day period, inhibitory behavior declined for neutral and self-relevant negative stimuli, in addition to overall performance. Figure 4 indicates a similar relationship for general negative words, though it was not statistically significant. There was no relationship between acute stress and inhibition for participants with inconsistent sleep schedules. Previous literature suggests that inhibition is often negatively impacted by stress (Killgore, 2010; Sandi, 2013; Taillard et al., 2021) and our results partially support these findings. However, sleep/wake regularity may be an important moderator that has not been fully considered in this context, suggesting that the relationship between sleep, stress, and cognitive inhibition may be more nuanced.

Previous literature examining the influence of stress on inhibitory control has focused primarily on stress induction paradigms (Starcke et al., 2016; Shields et al., 2019) and results suggest that exposure to a single acute stressor leads to reduced cognitive inhibition. In this study, we examined the cumulative impact of acute stress exposures as well as the impact of daily stress intensities on sleep and inhibition performance. While stress intensity was correlated with Stroop response times for both negative and self-relevant negative words, it did not modulate inhibitory performance after controlling for other variables. However, the number of stress exposures across the 21-day study did. First, those with the lowest sleep/wake regularity also reported the highest acute stress exposures. Here, those with low sleep/wake regularity reported acute stressor exposures on ~18% of days in the study vs. only ~6% of days in the study for those with consistent sleep/wake rhythms. Yet, those with reduced sleep/wake regularity did not see significant changes to response times on the Stroop task as stress exposures increased. This was in contrast to those with highly regular sleep/wake rhythms. For these participants, as stress exposures increased, their response times for both neutral and self-relevant negative words slowed. Given this pattern of results, one interpretation could be that individuals with highly regular sleep/wake rhythms are more deliberate under the influence of stress, which may account for their slowed performance. However, no interactions between stress and regularity on accuracy were found for any word type. This suggests that greater deliberation likely did not induce greater accuracy with increasing acute stress exposures.

Importantly, research has suggested that regular exposure to stress can dysregulate the psychological and physiological response to stress, resulting in blunted or exacerbated cardiovascular (Ferketich and Binkley, 2005) and hormonal responses (Kyrou and Tsigos, 2009; Holsboer and Ising, 2010) and atypical or maladaptive cognitive behaviors (Ouhmad et al., 2023). The findings here suggest that those with low sleep/wake regularity may have a blunted response to stress exposures given they did not emerge with the expected inhibitory performance deficits. Indeed, for those with an irregular sleep/wake schedule, there was no change in performance regardless of stress exposure. These data may indicate that sleep regularity helps to preserve our body's natural response to stress. Alternatively, stress-related vigilance may have benefited performance outcomes for individuals with lower sleep regularity given they also experienced more stress exposures. The data here cannot rectify these competing hypotheses, and as such, more data is needed. Additionally, stress and sleep factors often exhibit bidirectionality, with studies indicating that daily positive experiences can impact nightly sleep duration and quality, and that nightly sleep can predict next-day emotional wellbeing and the odds of stress exposure (Sin et al., 2017). Research has also implicated that executive function may serve to shape stress exposure and sleep behavior (O'Leary et al., 2017; Plieger and Reuter, 2020; Niu and Snyder, 2023). Additional studies are needed to better determine causality among these interdependent factors.

Previous literature indicated interactions between sleep and affect on inhibition where responding would be biased toward negative stimuli following poor sleep or sleep loss (Tempesta et al., 2010; Anderson and Platten, 2011; Lee et al., 2022). Here, we found a bias toward self-relevant negative words for those with more regular sleep/wake schedules, but no relationships with TST, SOL, or sleep quality. Importantly, previous studies included some form of experimental sleep deprivation, and we did not manipulate sleep here. While there were no statistically significant differences in TST between those with more vs. less sleep regularity, those in the bottom 10% of sleep regularity reported sleeping 36 min less than the top 10% and got around 20 min less than the recommended 7 h of sleep for their age range (Watson et al., 2015). Compared to a night of total sleep deprivation, this relatively minor sleep loss still resulted in faster response times for self-relevant negative words that is aligned with previous studies (Killgore, 2010; Tempesta et al., 2010, 2018; Watling et al., 2017). Additionally, acute stress exposures interacted with sleep/wake regularity and came at a cost to inhibition for both neutral and negative affective probes for those participants with regular sleep/wake timing. Importantly, we found no impact of acute stress exposure or stress intensity on working memory or cognitive flexibility. This stands in contrast to prior literature which indicated that acute stress in particular can result in worse outcomes for memory and flexibility (Sandi, 2013; Shields et al., 2016). Our data suggests instead that cognitive inhibition may be one executive function particularly sensitive to the combined impacts of stress and sleep.

## Limitations

This study is limited by its use of subjective reports of sleep and stress experiences. It is possible that participants' reported sleep times were not representative of their actual sleep schedules. Clinical mental health diagnoses and cognitive status were not recorded or screened, nor was medication use, and this could have influenced our results. Participants were not prompted to report their typical sleep times prior to entry into the study, however, inclusion criteria required 21-days of completed sleep diaries which provided us with a reliable estimate of habitual sleep patterns. A number of people from different backgrounds e.g., nationalities and ethnicities, participated in this study, however this was a sample of convenience with no active recruitment. As such, our sample was similar to other research studies and emerged as primarily White, from the U.S., cisgender, and male. Additionally, we delivered the cognitive tasks using a mobile app while people lived their daily lives. This can be considered a strength. However, this methodological approach may have also impaired our ability to validly assess cognitive performance. Participants completed these tasks in uncontrolled environments unlike controlled lab environments where these measures are typically assessed. Typical levels of stress were not collected prior to study participation, and our assessment of acute stress and stress intensities were evaluated with single questions. The forced-choice nature of these assessments precluded us from collecting data on current feelings of stress for participants who reported an acute stressor that day. Additionally, though we probed temporally-relevant stress events differently from pervasive feelings of stress and overwhelm in our daily surveys, we did not differentiate stressor cause or type which may limit our results. Future studies would benefit from comprehensive assessments of sleep and stress that include objective measurement techniques.

## Conclusion

This study utilized a unique daily diary approach to assess the individual and combined influences of sleep, sleep/wake regularity, and stress on executive functions. Acute stress events uniquely decreased inhibition of neutral and self-relevant negative stimuli for participants with highly regular sleep schedules, while no impacts were found for cognitive flexibility or working memory. Our results indicate the interdependency of sleep, stress, and executive function and underscore the need for future studies that disentangle causal relationships between these factors.

## Data availability statement

The original contributions presented in the study are publicly available. This data can be found here: https://osf.io/kj6pm.

## Ethics statement

The studies involving humans were approved by University of California, San Francisco (UCSF) IRB. The studies were conducted in accordance with the local legislation and institutional requirements. The participants provided their written informed consent to participate in this study.

## Author contributions

GG: Writing – original draft, Writing – review & editing. AS: Writing – review & editing. FD: Writing – original draft. AC: Writing – review & editing. WM: Writing – review & editing. LW: Writing – original draft, Writing – review & editing.
