# Supplementary material for: Sleep/wake regularity influences how stress shapes executive function
Source: Front Sleep. 2024 Apr 3;3:1359723. doi: 10.3389/frsle.2024.1359723 (PMC12713908; doi:10.3389/frsle.2024.1359723)
Supplement: Supplementary file 1 [file Table_1.DOCX]

**Supplementary Tables**

Table S.1: Emotional Stroop Word Lists

| Word List 1 | Word List 2 | Word List 3 | Word List 4 | Word List 5 | Category |
| --- | --- | --- | --- | --- | --- |
| \| quart \| \| --- \| \| locker \| \| paper \| \| egg \| \| method \| \| circle \| \| museum \| \| street \| \| unit \| \| fork \| \| injury \| \| coffin \| \| funeral \| \| sick \| \| terrified \| \| gun \| \| stroke \| \| destroy \| \| abuse \| \| bloody \| \| stupid \| \| lifeless \| \| tense \| \| inadequate \| \| obnoxious \| \| fake \| \| helpless \| \| disgraced \| \| incompetent \| \| disloyal \| | \| windmill \| \| --- \| \| quiet \| \| sphere \| \| finger \| \| plant \| \| tower \| \| lamb \| \| arm \| \| prairie \| \| ink \| \| execution \| \| trapped \| \| deathbed \| \| cancer \| \| killer \| \| fatal \| \| violence \| \| murder \| \| terrorist \| \| massacre \| \| anxious \| \| mistake \| \| inferior \| \| hated \| \| annoying \| \| awkward \| \| ignorant \| \| ridiculed \| \| conceited \| \| ashamed \| | \| hay \| \| --- \| \| humble \| \| moment \| \| door \| \| barrel \| \| jug \| \| column \| \| utensil \| \| taxi \| \| table \| \| afraid \| \| prison \| \| ambulance \| \| slave \| \| assault \| \| tumor \| \| torture \| \| crime \| \| tornado \| \| cruel \| \| troubled \| \| misfit \| \| betray \| \| embarrassed \| \| insecure \| \| stutter \| \| careless \| \| lonely \| \| failure \| \| foolish \| | \| banner \| \| --- \| \| chin \| \| pencil \| \| cabinet \| \| cord \| \| bathroom \| \| cork \| \| basket \| \| golfer \| \| inhabitant \| \| surgery \| \| deformed \| \| brutal \| \| hazard \| \| emergency \| \| accident \| \| captive \| \| intruder \| \| poison \| \| toxic \| \| ridiculous \| \| mocked \| \| insult \| \| Inept \| \| opinionated \| \| selfish \| \| coward \| \| idiotic \| \| nervous \| \| fraud \| | \| machine \| \| --- \| \| kerchief \| \| rain \| \| chair \| \| lamp \| \| hydrant \| \| vest \| \| patent \| \| horse \| \| hairpin \| \| bomb \| \| paralyzed \| \| burn \| \| mutilated \| \| disease \| \| pain \| \| trauma \| \| infection \| \| slaughter \| \| hostage \| \| sweaty \| \| rejected \| \| victim \| \| jealous \| \| pathetic \| \| shamed \| \| indecisive \| \| worthless \| \| criticized \| \| humiliated \| | \| neutral \| \| --- \| \| neutral \| \| neutral \| \| neutral \| \| neutral \| \| neutral \| \| neutral \| \| neutral \| \| neutral \| \| neutral \| \| negative \| \| negative \| \| negative \| \| negative \| \| negative \| \| negative \| \| negative \| \| negative \| \| negative \| \| negative \| \| self-relevant negative \| \| self-relevant negative \| \| self-relevant negative \| \| self-relevant negative \| \| self-relevant negative \| \| self-relevant negative \| \| self-relevant negative \| \| self-relevant negative \| \| self-relevant negative \| \| self-relevant negative \| |
